# MYB76 Inhibits Seed Fatty Acid Accumulation in Arabidopsis

**DOI:** 10.3389/fpls.2017.00226

**Published:** 2017-02-21

**Authors:** Shaowei Duan, Changyu Jin, Dong Li, Chenhao Gao, Shuanghui Qi, Kaige Liu, Jiangbo Hai, Haoli Ma, Mingxun Chen

**Affiliations:** College of Agronomy, Northwest A&F UniversityYangling, China

**Keywords:** MYB76, fatty acids, seeds, RNA sequencing, Arabidopsis

## Abstract

The MYB family of transcription factors is important in regulatory networks controlling development, metabolism and responses to biotic and abiotic stresses in Arabidopsis. However, their role in regulating fatty acid accumulation in seeds is still largely unclear. Here, we found that MYB76, localized in the nucleus, was predominantly expressed in developing seeds during maturation. The *myb76* mutation caused a significant increase in the amounts of total fatty acids and several major fatty acid compositions in mature seeds, suggesting that MYB76 functioned as an important repressor during seed oil biosynthesis. RNA sequencing and quantitative real-time PCR analysis revealed remarkable alteration of numerous genes involved in photosynthesis, fatty acid biosynthesis, modification, and degradation, and oil body formation in *myb76* seeds at 12 days after pollination. These results help us to understand the novel function of MYB76 and provide new insights into the regulatory network of MYB transcriptional factors controlling seed oil accumulation in Arabidopsis.

## Introduction

Fatty acids (FAs) and FA-derived complex lipids stored in the seed not only serve as the main source of nutrients for humans and livestock, but also facilitate successful seed germination and subsequent seedling establishment (Li et al., 2006; Graham, 2008). They can also be wildly used in industries (Lu et al., 2011) and serve as feedstock for biofuels production (Durrett et al., 2008). Therefore, understanding the role of key genes in the regulation of seed FA accumulation will provide great social and economic significances.

Transcriptional regulation, one of multi-level regulatory networks, is essential for the biosynthesis of seed FAs. Extensive studies have discovered that many transcription factors (TFs) control seed FA accumulation in Arabidopsis. WRINKLED1 (WRI1) promotes seed FA biosynthesis by directly or indirectly regulates some enzymes involved in late glycolysis and plastidial FA biosynthetic network (Focks and Benning, 1998; Cernac and Benning, 2004; Baud et al., 2007; Baud and Lepiniec, 2009; Maeo et al., 2009; Sanjaya et al., 2011). Activation of *LEAFY COTYLEDON1* (*LEC1*) results in the increased expression of many genes contributing to FA biosynthesis, thereby promoting the accumulation of major FA species (Mu et al., 2008). LEC1-LIKE (L1L) closely related to LEC1 also plays an important positive role in seed FA biosynthesis (Kwong et al., 2003; Mu et al., 2008; Baud and Lepiniec, 2010; Tan et al., 2011). Induction of *LEC2* activates the expression of genes involved in oil biosynthesis, which in turn trigger oil deposition in rosette leaves (Santos Mendoza et al., 2005; Braybrook et al., 2006). FUSCA 3 (FUS3) promotes oil deposition by positively regulating the expression of genes involved in photosynthesis and FA biosynthesis (Wang et al., 2007; Yamamoto et al., 2010; Zhang et al., 2016). GLABRA 2 (GL2) inhibits seed oil biosynthesis partly by influencing the formation of mucilage in the seed coat (Shi et al., 2012). BASIC LEUCINE ZIPPER TF 67 (bZIP67) regulates seed α-linolenic acid biosynthesis by directly binding to G-boxes in the *FATTY ACID DESATURASE 3* (*FAD3*) promoter (Mendes et al., 2013). TRANSPARENT TESTA 8 (TT8) represses seed FA deposition by targeting *LEC1, LEC2*, and *FUS3* (Chen et al., 2014). TRANSPARENT TESTA GLABRA 1 (TTG1) negatively regulates seed FA accumulation by indirectly repressing several FA biosynthesis genes (Chen et al., 2015).

As one of the largest family genes, MYB TFs are important in controlling development, metabolism and responses to biotic and abiotic stresses in all eukaryotes (Dubos et al., 2010). To the best of our knowledge, several MYB TFs play an important role in seed FA accumulation in Arabidopsis. MYB123 (TT2) inhibits seed FA biosynthesis by targeting *FUS3* (Chen et al., 2012b; Wang et al., 2014). MYB118 negatively regulates FA biosynthesis in the endosperm by repressing maturation-related genes (Barthole et al., 2014). MYB96 promotes the accumulation of very long chain fatty acids (VLCFAs; C ≥ 20) by directly regulating *FATTY ACID ELONGATION1* (*FAE1*) to during seed maturation (Lee et al., 2015). MYB89 inhibits seed FA accumulation by regulating *WRI1, L1L*, and key genes for FA biosynthesis (Li et al., 2017). However, the role of MYB TFs in regulating FA accumulation in seeds is still largely unknown and thus requires further exploration.

As one member of MYB TFs, MYB76 has been previously found to promote the accumulation of aliphatic glucosinolates in Arabidopsis leaves and seeds (Sønderby et al., 2007; Gigolashvili et al., 2008; Salles et al., 2013). In the present study, we found that MYB76, localized in the nucleus, is highly expressed in developing seeds during seed maturation in Arabidopsis. We demonstrated that MYB76 regulates numerous genes helpful for FA accumulation during seed maturation. Our results suggested that MYB76 functions as a negative TF of seed FA biosynthesis during seed maturation.

## Materials and methods

### Plant materials and growth conditions

Arabidopsis ecotype Col-0 was used as the wild type control. The mutants including *myb76-1* (SALK_096949) and *myb76-2* (SALK_055242C) were in the Col-0 background, and the genotyping primers are listed in Table S1. All plants for FA determinations were grown at the same time in the same chamber under long-day conditions (16-h light/8-h dark) at 22°C. The overhead light intensity was 160 μmol·m^−2^·s^−1^, as detected at the middle region of the plant.

### Morphological observation of mature seeds

Seeds were collected from the siliques at the basal part of a major inflorescence. Mature seeds from each line were randomly selected and photographed using an OLYMPUS SZ 61 stereomicroscope.

### Seed FA determination

The seeds for FA determination were harvested from the lower part of the main stem of 16 individual plants grown in different pots arranged randomly within one of three blocks. Seed FAs were extracted and analyzed as previously reported in detail (Poirier et al., 1999; Chen et al., 2012a). In brief, total FAs were converted to FA methyl esters in methanol solution containing 1 M HCl for 2 h at 80°C. FAs in seeds were subsequently measured using a gas chromatograph (GC-2014; Shimadzu).

### Analysis of seed storage proteins by SDS-PAGE

Analysis of seed storage proteins was performed as previously described (Chen et al., 2014, 2015). In brief, 1 mg of mature dry seeds was homogenized with 25 μL of extraction buffer (100 mM Tris-HCl, pH 8.0, 0.5% [v/v] SDS, 10% [v/v] glycerol, and 20% [v/v] 2-mercaptoethanol) using a microglass pestle and mortar. After transient centrifugation and subsequent boiling for 5 min, the extract was centrifuged again and the supernatant was used for SDS-PAGE analysis.

### RNA-Seq and data analyses

The flowers of the wild type (Col-0) and *myb76-2* plants were tagged with different colored threads to indicate days after pollination (DAP). Only the developing seeds from the siliques on the primary shoots of 80 individual plants for each genotype in one biological replicate, which were grown in different pots arranged randomly, were used for RNA-Seq experiment. Two independent biological replicates from two different plantings were performed for the wild type and *myb76-2* in the RNA-Seq experiment. The following analysis was conducted using the services of GENE DENOVO Inc. (http://www.genedenovo.com/) following the standard protocol (http://www.genedenovo.com/product/41.html). The Excel add-in for significance analysis of RNA-Seq was used to identify DEGs between the wild type and *myb76-2*. The DEGs were functionally classified using the biological process category of Arabidopsis Gene Ontology (GO) (http://www.geneontology.com). The DEGs with log_2_ ratios of ≥1.00 or ≤ −1.00 (only GO Slim IDs with *P* ≤ 0.05 and *FDR* ≤ 0.05) are listed in Tables S2, S3.

### Gene expression analysis

Total RNA samples were isolated from vegetative tissues or developing seeds using the MiniBEST Plant RNA Extraction Kit (TaKaRa) and reverse transcribed using PrimerScript RT (TaKaRa). The developing seeds were from at least 12 individual plants grown in different pots arranged randomly, and three independent biological replicates from three different plantings were used for the expression analysis. Quantitative real-time (qRT)-PCR was conducted for three biological replicates by using SYBR Green Master Mix (TaKaRa). The ubiquitously expressed Arabidopsis *EF1aA4* gene was used as an internal control. Primers used for qRT-PCR analysis are listed in Table S1.

## Results

### Analysis of *MYB76* expression pattern

According to the Arabidopsis eFP Browser, *MYB76* is predominantly present in mature pollen, hypocotyl, and developing seeds, particularly at the late stages of seed development, indicating that it may regulate seed-related traits. Therefore, MYB76 was selected to investigate the role of MYB TFs in the regulation of seed FA accumulation.

**Figure 1:**
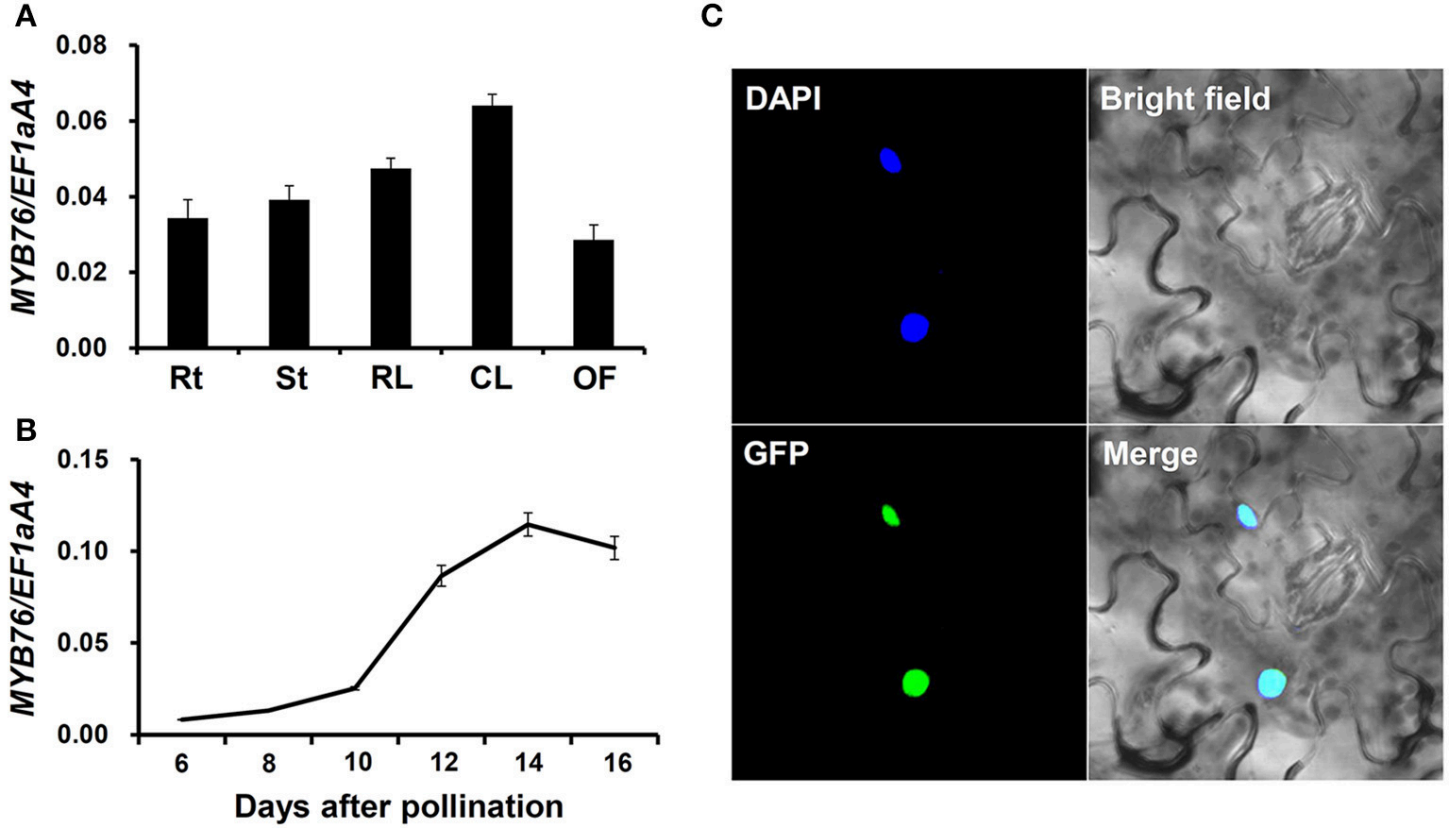
**Analysis of *MYB76* expression pattern. (A)** qRT-PCR analysis of *MYB76* expression in various tissues of the wild type (Col-0). Rt, roots; St, stems; RL, rosette leaves; CL, cauline leaves; OF, open flowers. **(B)** qRT-PCR analysis of *MYB76* expression in developing seeds of the wild type. **(C)** Subcellular localization of MYB76 protein fused with GFP (*35S:MYB76-GFP*) in tobacco (*Nicotiana benthamiana*) leaves. DAPI, fluorescence of 4′,6-diamino-2-phenylindole; Merge, merge of GFP, DAPI, and bright field images. All the qRT-PCR results were normalized against the expression of *EF1aA4* as an internal control. Error bars denote SD.

We measured *MYB76* expression in various tissues of the wild type plants using qRT-PCR. As shown in Figures 1A,B, *MYB76* was widely expressed in various tissues, and was also predominantly present in developing seeds. The *MYB76* transcript level was higher in rosette and cauline leaves than it was in roots, stems, and open flowers (Figure 1A). During seed development, *MYB76* expression constantly increased at 8 DAP to the maximal level at 14 DAP, and slightly decreased afterwards (Figure 1B). This suggested that *MYB76* regulates seed oil accumulation mainly occurring at the maturation stage in Arabidopsis seeds (Baud et al., 2002, 2008; Fait et al., 2006; Graham, 2008; Baud and Lepiniec, 2009).

We also investigated the subcellular localization of MYB76 in tobacco leaves using the green fluorescent protein (GFP) fusion construct, *35S:MYB76-GFP*. MYB76-GFP was specifically localized in the nucleus (Figure 1C), thereby further demonstrating that MYB76 functions as a TF.

### MYB76 inhibits FA biosynthesis in seeds

To explore MYB76 effect on seed FA accumulation, two T-DNA insertion mutants SALK_096949 and SALK_055242C in the Columbia (Col-0) background, which have been previously designated *myb76-1* and *myb76-2*, respectively (Sønderby et al., 2007), were backcrossed thrice with Col-0 and then used for FA analysis in this study (Figure S1).

**Figure 2:**
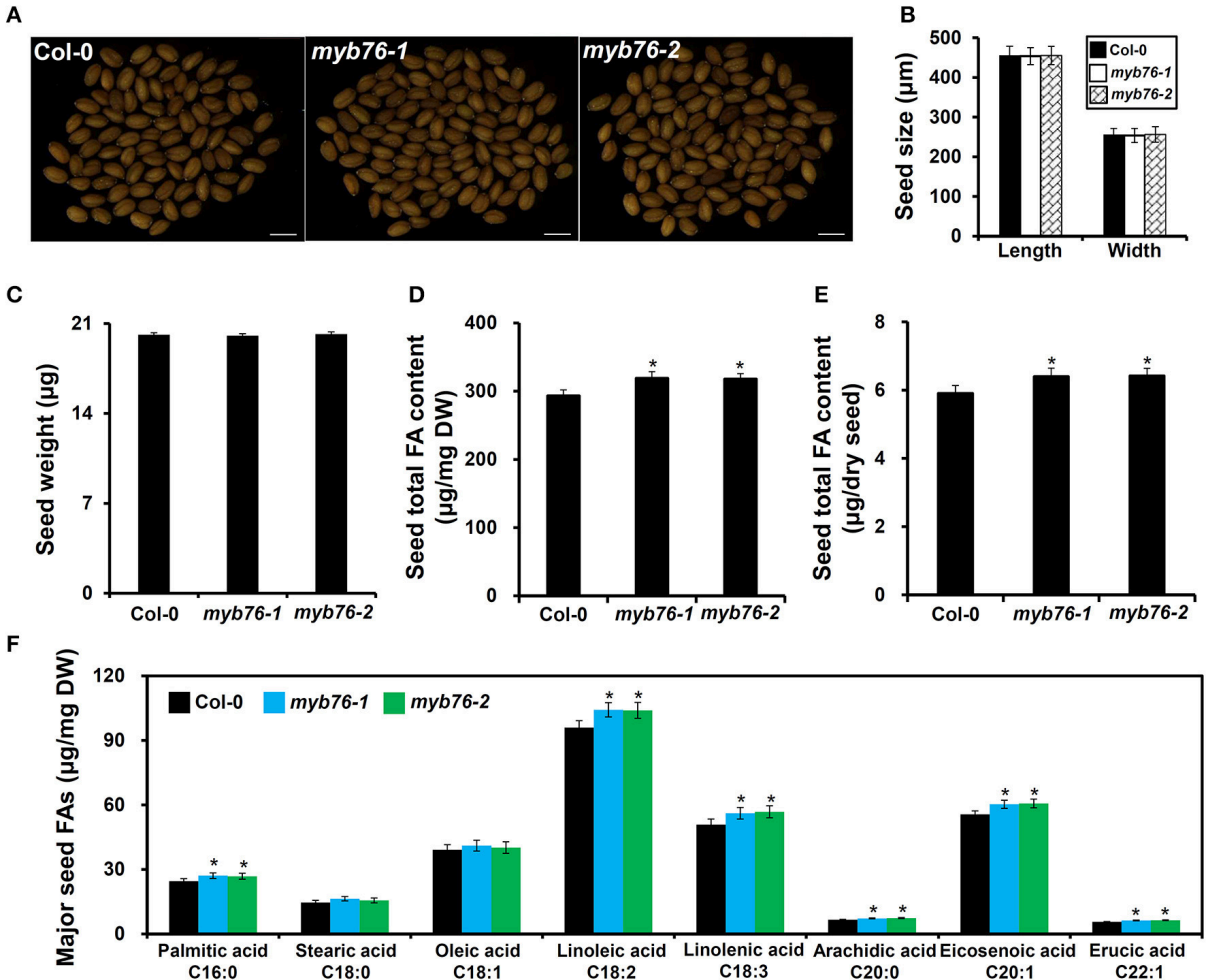
**Characterization of *MYB76* function on seed FA biosynthesis. (A)** Microscopic observation of mature seeds randomly selected from the wild type (Col-0) and *myb76* plants. Bars: 500 μm. **(B)** Quantitative comparisons of seed size (length and width) between the wild type (Col-0) and *myb76* plants. **(C)** Quantitative comparison of dry weight of seeds between the wild type (Col-0) and *myb76* plants. **(D)** Comparison of seed total FA content (μg/mg) between the wild type (Col-0) and *myb76* plants. **(E)** Comparison of seed total FA content (μg/dry seed) between the wild type (Col-0) and *myb76* plants. **(F)** Comparison of contents of major seed FA compositions between the wild type (Col-0) and *myb76* plants. Asterisks indicate significant differences in the seed total FA content **(D,E)** and the contents of major seed FA compositions **(F)** compared to that in the wild type (two-tailed paired Student's *t*-test, *P* ≤ 0.05). Values are means ± SD (*n* ≥ 3). DW, dry weight. Error bars denote SD.

The results showed that the level of total FAs was much higher in *myb76* than in the wild type seeds (Figures 2D,E). Consistently, the contents of major FA compositions except for C18:0 and C18:1 were also considerably higher than in the wild type seeds (Figure 2F). However, no obvious differences were observed in several seed morphological traits including seed coat color (Figure 2A), seed size and dry weight (Figures 2B,C), and the content and components of seed storage proteins (Figure S2) between mature seeds of the wild type and *myb76* plants. These results suggested that MYB76 represses seed FA accumulation during seed maturation.

### Differentially expressed genes in developing seeds at 12 Dap between wild type and *MYB76-2* plants

We elaborately harvested developing seeds at 12 DAP to compare the expressional profiles between the wild type and *myb76-2* plants. Previous studies have showed that 12 DAP is the key stage for seed FA biosynthesis during seed maturation (Fait et al., 2006; Baud et al., 2008; Graham, 2008; Baud and Lepiniec, 2009; Chen et al., 2012b; Li et al., 2017). Thus, transcriptome analysis of developing seeds at 12 DAP would provide useful information on the downstream targets of MYB76 that contribute to FA accumulation, as well as facilitate a better understanding of the regulatory networks underlying MYB76-mediated seed FA biosynthesis.

RNA-Seq analysis identified 206 differentially expressed genes (DEGs), among which 99 were upregulated (Table S2) and 107 were downregulated (Table S3) in *myb76-2* developing seeds at 12 DAP. Consistent with the previous studies (Gigolashvili et al., 2007, 2008; Beekwilder et al., 2008; Sønderby et al., 2010; Salles et al., 2013), several genes including *CYP79F1, CYP83A1, BCAT4, MAM1*, and *MAM3* in the aliphatic glucosinolate biosynthetic pathway and *MYB28* were significantly induced in *myb76-2* than that of the wild type (Table S2). This suggested that the RNA-Seq experiment using *myb76-2* developing seeds at 12 DAP was properly conducted, and the RNA-seq result was reliable. Functional analysis showed that 7 (7.1%) of the unregulated genes and 12 (11.2%) of the downregulated genes were related to oil metabolism (Table 1; Tables S2, S3). Interestingly, two *GDSL-LIKE LIPASE* genes were significantly downregulated in *myb76-2* seeds (Table S3). Notably, 8 (8.1%), 11 (11.1%), and 8 (8.1%) of the unregulated genes were involved in photosynthesis, carbohydrate metabolism, and general protein metabolism, respectively (Table 1; Table S2). However, the expression of many genes contributing to oil accumulation did not change in *myb76-2* seeds in comparison with that of the wild type seeds (Table S4).

**Table 1:** **Functional classification of DEGs in developing seeds of *myb76-2* plants at 12 DAP**.

**Category**	**Upregulated DEGs**				**Downregulated DEGs**			
	**≥2**	**1–2**	**Total**	**Percentage**	** ≤ −2**	**−2 to −1**	**Total**	**Percentage**
**log**_2_ **ratio**								
**METABOLISM**								
Glucosinolate biosynthesis	5	0	5	5.1	0	0	0	0.0
Photosynthesis	3	3	6	6.1	0	1	1	0.9
Cell wall	0	1	1	1.0	0	2	2	1.9
Oil metabolism	3	4	7	7.1	2	10	12	11.2
Carbohydrate metabolism	2	9	11	11.1	3	4	7	6.5
Nucleic acid	0	2	2	2.0	3	2	5	4.7
Amino acid and protein	3	4	7	7.1	0	5	5	4.7
**GROWTH AND DEVELOPMENT**								
Leaf and root development	1	1	2	2.0	0	0	0	0.0
Shoot development	0	1	1	1.0	0	1	1	0.9
Embryo/seed development	1	0	1	1.0	0	3	3	2.8
Flower development	0	2	2	2.0	0	2	2	1.9
Cell growth	0	0	0	0.0	0	1	1	0.9
Hormone	1	5	6	6.1	2	3	5	4.7
Stress/defense response	4	4	8	8.1	2	23	25	23.4
**CELL REGULATION**								
Transcriptional regulation	2	4	6	6.1	0	0	0	0.0
Signaling transduction	3	0	3	3.0	0	2	2	1.9
Transport facilitation	3	3	6	6.1	0	4	4	3.7
Others	8	17	25	25.3	5	27	32	29.9

*Functional classification of DEGs was performed using the biological process category of Arabidopsis Gene Ontology (http://www.geneontology.com). Percentage (far-right column) refers to the ratio of genes of each functional category relative to total upregulated or downregulated DEGs identified in the RNA-seq experiment. The DEGs with log_2_ ratios greater than 1.00 or less than −1.00 (only GO Slim IDs with P ≤ 0.05 and FDR ≤ 0.05) are listed*.

In all, 6 genes related to transcriptional regulation were unregulated in *myb76-2* seeds (Table 1). No significant differences in the expression of the known master regulators of oil accumulation were observed between the wild type and *myb76-2* developing seeds at 12 DAP (Table S4).

### Confirmation of regulated genes affecting FA accumulation at different developmental stages in *MYB76-2* developing seeds

**Figure 3:**
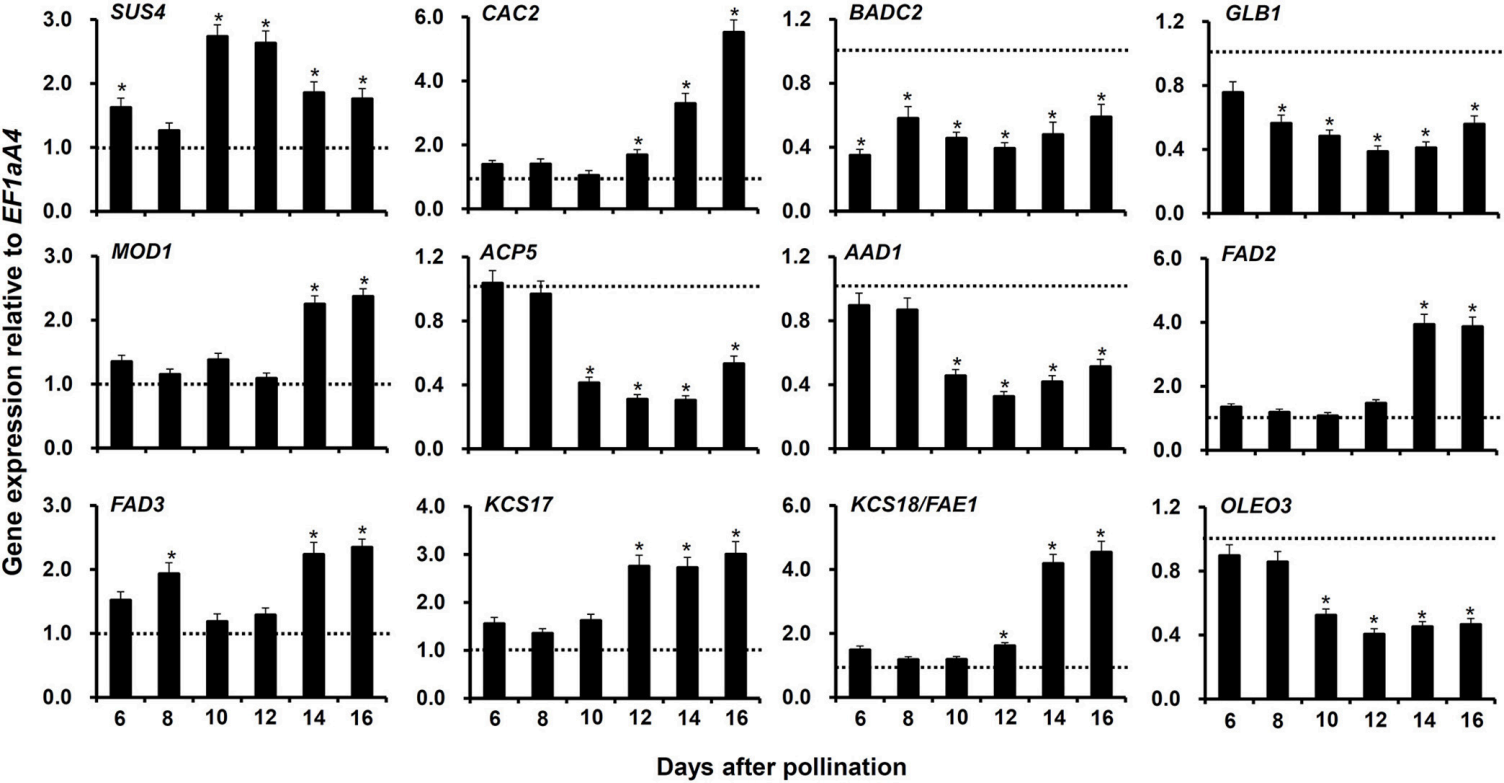
**Comparison of the expression of genes contributing to FA biosynthesis or modification in the developing seeds of the wild type (Col-0) and *myb76-2* plants**. RNA samples were extracted from developing seeds at different developmental stages and values are the means of two replicates, carried out using cDNA dilutions obtained from two independent RNA extractions. Results were estimated based on the expression levels of *EF1aA4*, set as the internal standard. The gene expression level in the wild type was set to one at each developmental stage. Two-tailed paired Student's *t*-test significance: *P ≤ 0.05. Error bars denote SD.

To verify the regulation of genes contributing to FA accumulation in *myb76-2* developing seeds at 12 DAP, and to extensively investigate potential genes involved in FA accumulation that are regulated by MYB76, we conducted qRT-PCR to compare the expression patterns at the seed maturation stages (6–16 DAP) between the wild type and *myb76-2* plants. First, we selected 7 highly regulated genes contributing to FA biosynthesis, which include *SUCROSE SYNTHASE 4* (*SUS4*), *BIOTIN ATTACHMENT DOMAIN CONTAINING 2* (*BADC2*), *GLNB1 HOMOLOG* (*GLB1*), *ACYL CARRIER PROTEIN 5* (*ACP5*), *ACYL-ACYL CARRIER PROTEIN DESATURASE 1* (*AAD1*), *3-KETOACYL-COA SYNTHASE 17* (*KCS17*), and *OLEOSIN3* (*OLEO3*), in *myb76-2* developing seeds at 12 DAP (Figure 3). The expression of *SUS4* was significantly higher at 10 and 12 DAP in the *myb76-2* seeds than that in the wild type seeds. Relative to that of the wild type, the *myb76* mutation caused a two-fold higher level of *KCS17* transcripts in developing seeds from 12 to 16 DAP. However, the expression of *BADC2* was much lower during seed maturation except for 8 and 16 DAP, and the expression of *GLB1* was also considerably lower from 8 to 16 DAP, in *myb76-2* than that of the wild type. The expression levels of other three genes including *ACP5, AAD1*, and *OLEO3* were all significantly lower in *myb76-2* than that of the wild type from 10 to 16 DAP.

Second, another 11 genes (Figure 3; Figure S3), the expression of which was not significantly changed in *myb76-2* developing seeds at 12 DAP (Table S4), were selected to explore whether these are regulated by MYB76 during seed maturation. No significant differences in the transcripts of 6 genes were observed at the seed maturation stage between the wild type and *myb76-2* plants (Figure S3), except that the expression of *CAC2, MOSAIC DEATH 1* (*MOD1*), *FATTY ACID DESATURASE 2* (*FAD2*), *FAD3*, and *KCS18*/*FAE1* was significantly increased in *myb76-2* developing seeds at 14 and 16 DAP (Figure 3).

**Figure 4:**
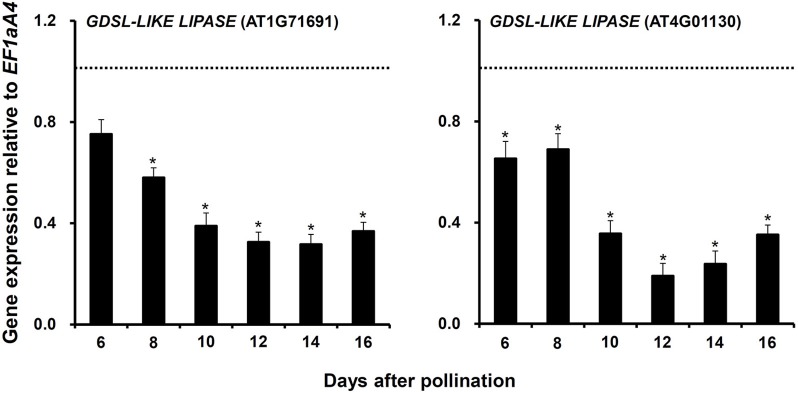
**Comparison of the expression of two *GDSL-LIKE LIPASE* genes in the developing seeds of the wild type (Col-0) and *myb76-2* plants**. RNA samples were extracted from developing seeds at different developmental stages and values are the means of two replicates, carried out using cDNA dilutions obtained from two independent RNA extractions. Results were estimated based on the expression levels of *EF1aA4*, set as the internal standard. The gene expression level in the wild type was set to one at each developmental stage. Two-tailed paired Student's *t*-test significance: *P ≤ 0.05. Error bars denote SD.

Third, two *GDSL-LIKE LIPASE* genes (AT1G71691 and AT4G01130), whose expressions were significantly downregulated in *myb76-2* developing seeds at 12 DAP (Table S3), were selected to investigate whether they are regulated by MYB76 at other stages during seed maturation. The results showed that the expression of the two genes was significantly decreased from 10 to 16 DAP and was always lower in *myb76-2* than that of the wild type (Figure 4).

Taken together, these findings indicated that MYB76 represses seed FA accumulation through regulating the expression of these genes during seed maturation.

## Discussion

Transcriptional regulation controlling seed FA biosynthesis in higher plants is still largely unclear to date. In this study, we found that the *myb76* mutation resulted in a considerable increase in the amounts of total FAs and several major FA compositions in mature seeds (Figures 2D–F). This, together with the observation of increased expression of *MYB76* in developing seeds at the mid-seed maturation stage (Figure 1B), indicated that MYB76 plays an important role in the regulation of seed oil accumulation in Arabidopsis.

The main storage compounds accumulated in Arabidopsis seeds mainly consist of oil and seed storage proteins, which are definitely derived from the photosynthetic products (Baud et al., 2008; Baud and Lepiniec, 2009). Sucrose serves as the major form in which photosynthetically assimilated carbon is transported in plants. The number of upregulated genes involved in the metabolism of photosynthesis and carbohydrates was higher than that of the downregulated genes in *myb76-2* seeds (Table 1; Tables S2, S3). Loss of function of *SUS4* results in a decrease in sucrose content in leaves, whereas the oil content is not altered (Bieniawska et al., 2007). *SUCROSE-PROTON SYMPORTER 1* (*SUC1*) as the energy-dependent sucrose/H+ symporter plays an important role in loading of sucrose into phloem companion cells (Wippel and Sauer, 2012). However, the amount and components of storage proteins in *myb76* seeds was not altered in comparison with the wild type (Figure S2). Besides, the *myb76* mutation caused the decreased content of aliphatic glucosinolates (Sønderby et al., 2007; Gigolashvili et al., 2008; Salles et al., 2013). The higher expression of *SUS4, SUC1*, and other upregulated photosynthetic genes (Figure 3; Table S2) should have a potentiating effect on the production and transport of photosynthetic products in *myb76* developing seeds, thereby providing more carbon source for FA biosynthesis in *myb76* mature seeds.

The precursor acetyl-coenzyme A (acetyl-CoA) for FA synthesis is produced from sucrose through the glycolysis, the oxidative pentose phosphate pathway (OPPP), and the RuBisCO shunt. The formation of malonyl-CoA from acetyl-CoA is catalyzed by HtACCase in the FA biosynthetic pathway (Wang et al., 2007; Baud et al., 2008; Santos-Mendoza et al., 2008; Baud and Lepiniec, 2009; Li et al., 2017). HtACCase acts as the key switch that monitors the overall flux of FA biosynthesis in plastids (Mu et al., 2008). BADC2 interacts with and inhibits HtACCase activity in Arabidopsis (Salie et al., 2016). *GLB1* encodes a PII protein that inhibited HtACCase activity in a dose-dependent manner (Baud et al., 2010; Feria Bourrellier and Haselkorn, 2010). *CAC2* as HtACCase subunits was significantly upregulated and *BADC2* was considerably downregulated during *myb76-2* seed maturation (Figure 3). In the case of sufficient photosynthetic products, more active HtACCase should result in increased FA accumulation in *myb76* seeds. However, further studies are required to explore how MYB76, BADC2, and PII regulate the expression of *CAC2* during seed maturation. The *MOD1* gene encodes an enoyl-acyl carrier protein (ACP) reductase, and total oil content was markedly reduced in *mod1* plants (Mou et al., 2000). The considerable upregulation of *MOD1* in *myb76* developing seeds (Figure 3) should accelerate FA biosynthesis in *myb76* seeds. Previous studies showed that the depletion of oleosins in the seed oil body significantly reduced the seed oil content (Lu et al., 2006; Siloto et al., 2006). The expression of *OLEO3* was significantly decreased during *myb76* seed maturation (Figure 3; Table S3), however, the OLEO3 protein only accounts for less than 5% of oleosins (Chen et al., 1999; Lin et al., 2002). Therefore, the downregulation of *OLEO3*, to a maximum extent, might slightly repress seed FA deposition in *myb76* seeds. Interestingly, MYB76 greatly promoted the expression of two *GDSL-LIKE LIPASE* genes (AT1G71691 and AT4G01130) during seed maturation (Figure 4; Table S3). As the GDSL-type lipase gene, *SEED FATTY ACID REDUCER* (*SFAR*) inhibits seed FA accumulation by involving in FA degradation (Chen et al., 2012a). It can be deduced that the much lower expression of the two *GDSL-LIKE LIPASE* genes should greatly contribute to the higher content of FAs in *myb76* seeds (Figures 2D–F).

It is worth mentioning that the *GLB1* mutation significantly decreased the contents of VLCFAs and polyunsaturated FAs including C18:2 and C18:3 in mature seeds (Baud et al., 2010). Therefore, significantly increased amounts of major FA compositions examined except for C18:0 and C18:1 in *myb76* seeds (Figure 2F) was not due to the decreased expression of *GLB1* in *myb76* developing seeds. The alteration in the amounts of major FA species in *myb76* seeds might be the consequence of an accelerated rate of *de novo* FA biosynthesis. Moreover, this should be greatly caused by the decreased expression of *ACP5* and *AAD1*, and the increased expression of *FAD2, FAD3, KCS17*, and *KCS18* during *myb76-2* seed maturation. ACYL CARRIER PROTEINS (ACPs) are helpful for the biosynthesis of 16- and 18-C acyl groups by carrying the nascent acyl chains (Hannapel and Ohlrogge, 1988; Bonaventure and Ohlrogge, 2002). The loss of *AAD1* contained comparative C18:1 level with the wild type, however, ectopic expression of *AAD1* in *fab2* background rescued the lower amount of C18:1 (Kachroo et al., 2007). The lower expression of *ACP5* and *AAD1* might be the reason for the unaltered contents of C18:0 and C18:1 in *myb76* seeds (Figure 2F). FAD2 plays an essential role in the biosynthesis of polyunsaturated FAs (Okuley et al., 1994), and FAD3 is vital for C18:3 biosynthesis from phospholipids (Shah et al., 1997). Thus, the significant upregulation of *FAD2* and *FAD3* would separately promote the deposition of C18:2 and C18:3, which possibly in turn resulted in the unaltered accumulation of C18:0 and C18:1 in *myb76* seeds (Figure 2F). The *KCS* family genes are responsible for VLCFAs biosynthesis and facilitate the chain length extension of FAs from C18 to C20 and C22. The highly increased expression of *KCS17* and *KCS18*/*FAE1* could well explain why *myb76-2* seeds accumulated much more VLCFAs than the wild type (Figure 2F).

In summary, this study first discovered that a MYB TF, MYB76 represses seed FA accumulation by regulating the expression of genes involved in photosynthesis, FA biosynthesis, modification, and degradation, and oil body formation in Arabidopsis (Figures 3, 4; Tables S2, S3). Together with previous discoveries that the *myb76* mutation caused the decreased deposition of aliphatic glucosinolates in Arabidopsis seeds (Sønderby et al., 2007; Gigolashvili et al., 2008; Salles et al., 2013), *MYB76* can be used as a promising target to genetically manipulate *Brassica napus* to improve both the quantity and quality of FAs and decrease the glucosinolate accumulation in seeds.

## Author contributions

SD and CJ carried out the experiments. SD and DL analyzed the data. CG, SQ, and KL assisted with doing the experiments. MC conceived and designed the experiments. SD and MC wrote the manuscript. JH and HM helped to draft the manuscript and revise the manuscript. All authors read and approved the final manuscript.

### Conflict of interest statement

The authors declare that the research was conducted in the absence of any commercial or financial relationships that could be construed as a potential conflict of interest.

## Supplementary material

Figure S1 **Molecular identification of the *myb76* mutation. (A)** Structure of the *MYB76* gene showing the position of the T-DNA insertions in SALK_096949 (*myb76-1*) and SALK_055242C (*myb76-2*) mutants. Black boxes represent exons while open boxes stand for introns. **(B)** PCR-based genotyping of the two mutants. LP and RP refer to the *MYB76* gene specific primers and BP refers to T-DNA right-border primer given in Table S1. Error bars denote SD.

Figure S2 **Comparative analysis of seed storage protein components from the wild type and *myb76* plants**. The same volume of protein extracts from each genotype was loaded onto the 15% SDS polyacrylamide gel and stained. The locations of the major storage proteins including 12S and 2S are indicated on the left. The Lanes in sequence from left to right are wild type (Col-0), *myb76-1*, and *myb76-2*.

Figure S3 **Comparison of the expression of genes controlling FA biosynthesis in the developing seeds of the wild type (Col-0) and *myb76-2* plants**. RNA samples were extracted from developing seeds at different developmental stages and values are the means of two replicates, carried out using cDNA dilutions obtained from two independent RNA extractions. Results were estimated based on the expression levels of *EF1aA4*, set as the internal standard. The gene expression level in the wild type was set to one at each developmental stage. No significant differences were observed between the wild type and *myb76-2* plants (two-tailed paired Student's *t*-test, *P* ≤ 0.05). Error bars denote SD.

Table S1 **Primers used in this study**.

Table S2 **A list of upregulated genes in developing seeds of *myb76-2* plants at 12 DAP**.

Table S3 **A list of downregulated genes in developing seeds of *myb76-2* plants at 12 DAP**.

Table S4 **A list of regulatory and structural genes for oil biosynthesis whose expressions were not altered in developing seeds of *myb76-2* plants at 12 DAP**.
