# Supplementary figures and images for: MYB76 Inhibits Seed Fatty Acid Accumulation in Arabidopsis
Source: Front Plant Sci. 2017 Feb 21;8:226. doi: 10.3389/fpls.2017.00226 (PMC5318433; doi:10.3389/fpls.2017.00226)

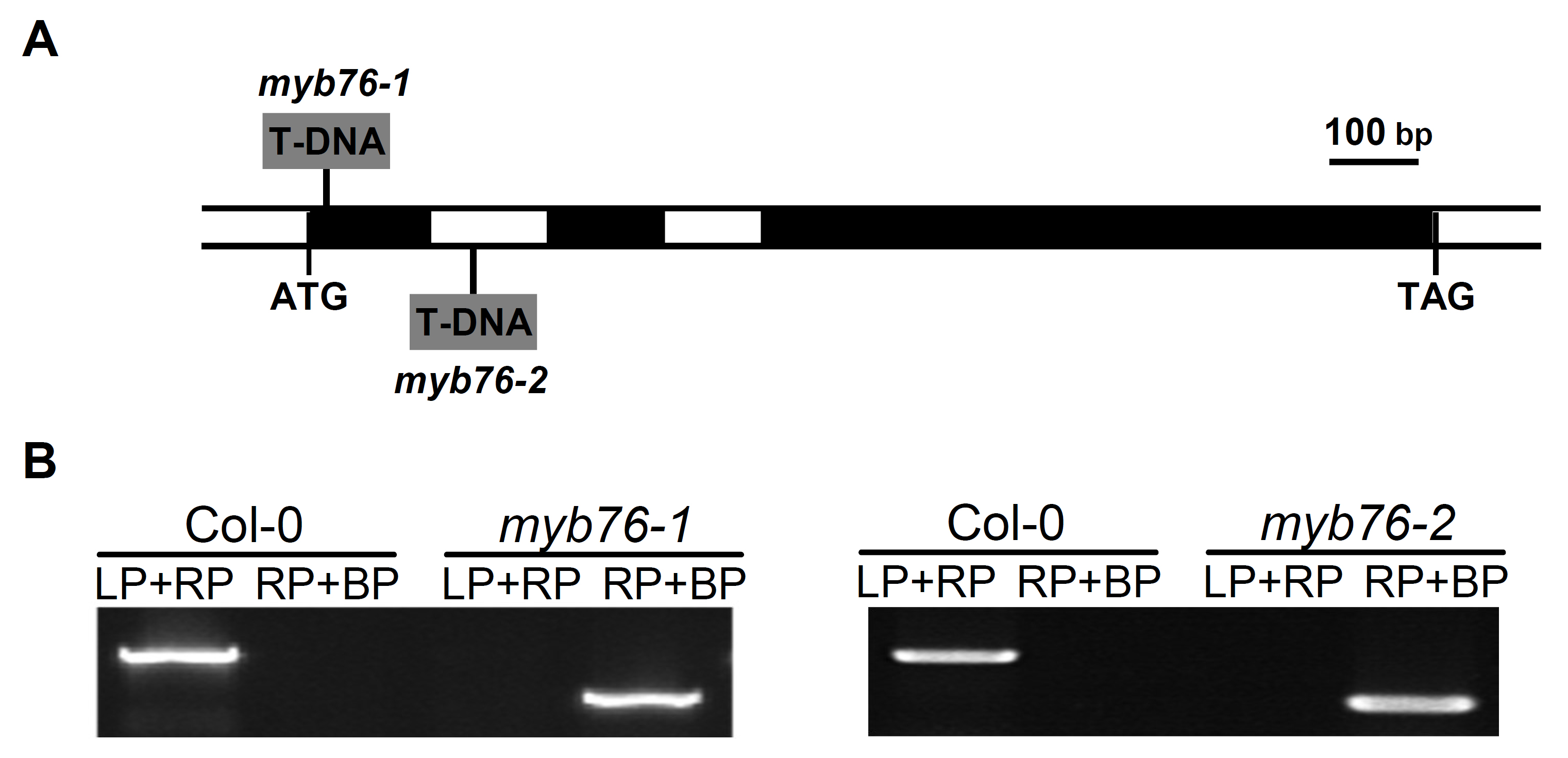

Supplement: Figure S1 — Molecular identification of the myb76 mutation. (A) Structure of the MYB76 gene showing the position of the T-DNA insertions in SALK_096949 (myb76-1) and SALK_055242C (myb76-2) mutants. Black boxes represent exons while open boxes stand for introns. (B) PCR-based genotyping of the two mutants. LP and RP refer to the MYB76 gene specific primers and BP refers to T-DNA right-border primer given in Table S1. Error bars denote SD. [file Image1.JPEG]

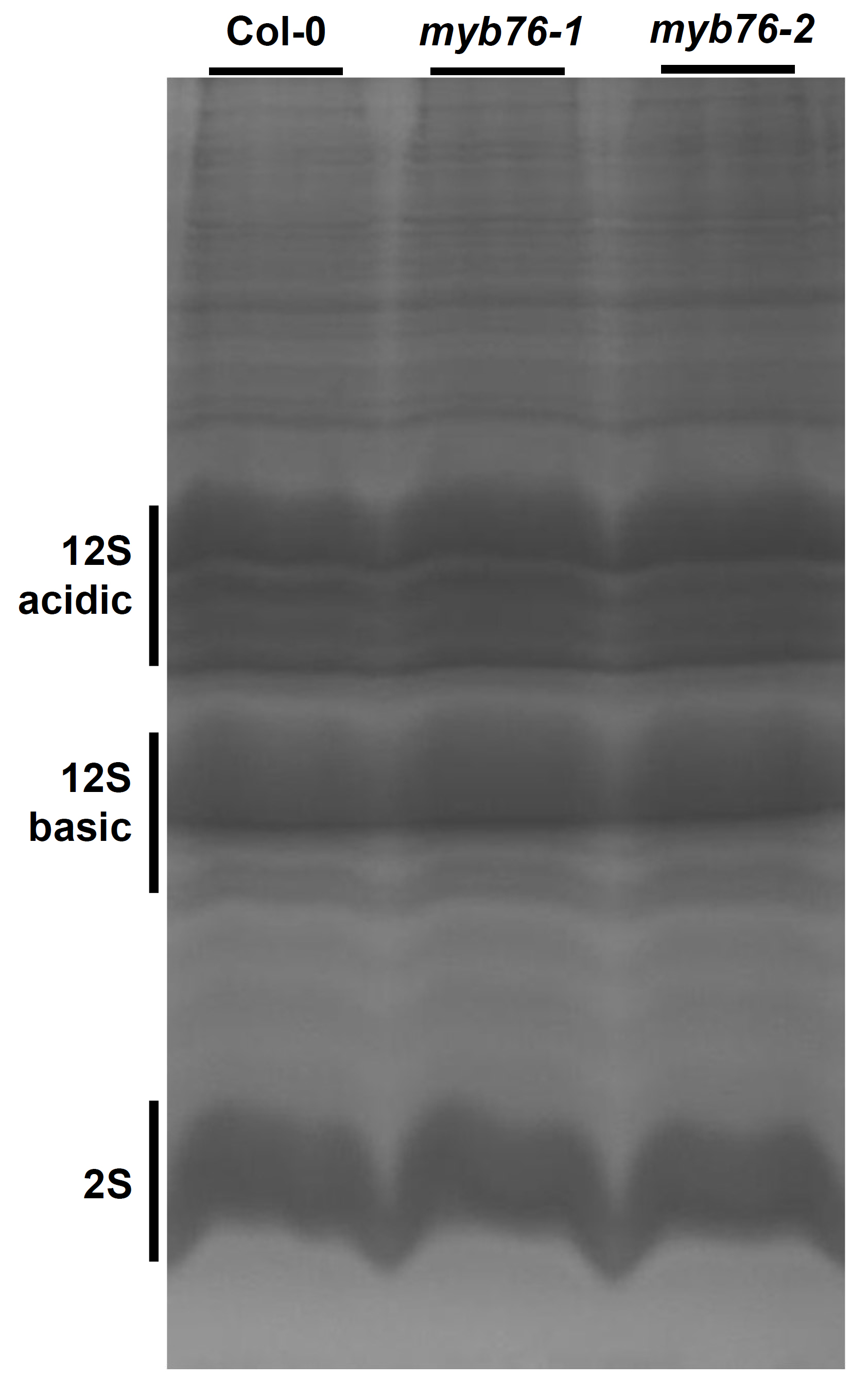

Supplement: Figure S2 — Comparative analysis of seed storage protein components from the wild type and myb76 plants. The same volume of protein extracts from each genotype was loaded onto the 15% SDS polyacrylamide gel and stained. The locations of the major storage proteins including 12S and 2S are indicated on the left. The Lanes in sequence from left to right are wild type (Col-0), myb76-1, and myb76-2. [file Image2.JPEG]

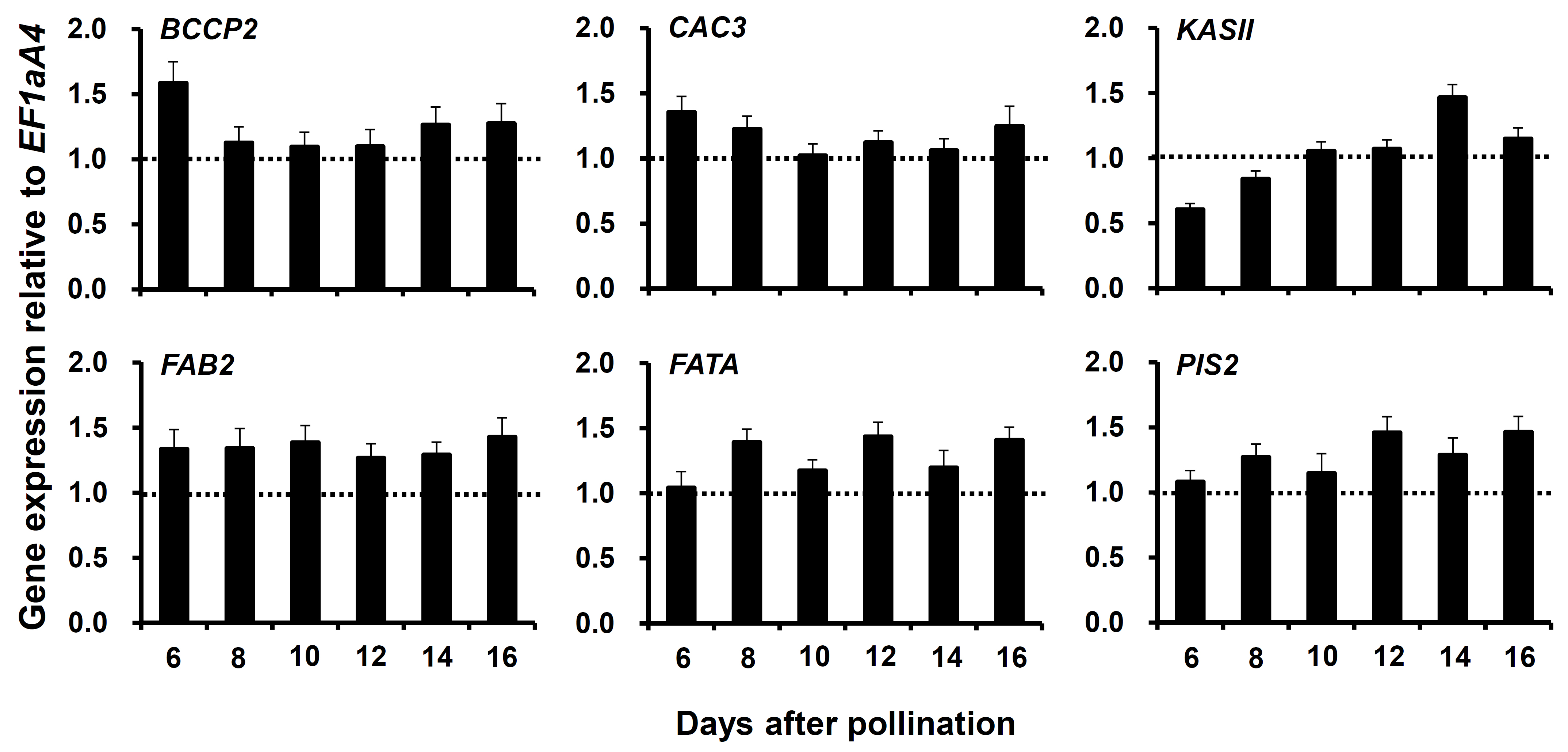

Supplement: Figure S3 — Comparison of the expression of genes controlling FA biosynthesis in the developing seeds of the wild type (Col-0) and myb76-2 plants. RNA samples were extracted from developing seeds at different developmental stages and values are the means of two replicates, carried out using cDNA dilutions obtained from two independent RNA extractions. Results were estimated based on the expression levels of EF1aA4, set as the internal standard. The gene expression level in the wild type was set to one at each developmental stage. No significant differences were observed between the wild type and myb76-2 plants (two-tailed paired Student's t-test, P ≤ 0.05). Error bars denote SD. [file Image3.JPEG]
